# Eutectogels: Recent Advances, Design Strategies, and Emerging Applications in Biotechnology

**DOI:** 10.3390/gels11121013

**Published:** 2025-12-17

**Authors:** Liane Meneses, Ana Rita Jesus

**Affiliations:** Laboratório Associado Química Verde-REQUIMTE, Faculdade de Ciências e Tecnologia, Universidade Nova de Lisboa, 2829-516 Caparica, Portugal; lp.meneses@fct.unl.pt

**Keywords:** natural deep eutectic systems, eutectogels, hydrogels, biotechnology

## Abstract

Eutectogels, obtained from the combination of deep eutectic systems (DESs) or natural deep eutectic systems (NADESs) with polymers, represent a new class of sustainable soft materials. Combining the tunable properties of DESs, such as low volatility, ionic conductivity, and biocompatibility, with the structural integrity of gels, these materials can be designed to have improved mechanical flexibility, self-healing ability, and environmental stability. Recent research focused on understanding how the composition of DESs, polymer type, or crosslinking mechanisms influence the physicochemical behavior and performance of eutectogels. Advances in this field enabled their use in diverse biotechnological applications, particularly in drug delivery, transdermal systems, wound healing, and tissue engineering, where they demonstrate improved biofunctionality and adaptability compared to traditional hydrogels. Nevertheless, challenges related to scalability, reproducibility, long-term stability, and toxicity must be addressed to reach their full potential. Progress in this area relies on multidisciplinary efforts between green chemistry, materials science, and bioengineering. Overcoming these hurdles could allow eutectogels to evolve from academic concepts into a new generation of sustainable, high-performance soft materials with broad applicability in the biotechnology field.

## 1. Introduction

Gels are soft materials composed of a three-dimensional (3D) polymer network swollen by a liquid phase, exhibiting properties that lie between solids and liquids [1]. Due to their high-water content and tunable mechanical characteristics, gels have gathered significant interest across a wide range of disciplines, including biomedical engineering, materials science, and soft robotics [2,3,4]. Conventional hydrogels paved the way for a broad array of applications; however, they are often limited by factors such as poor mechanical strength, low thermal stability, or incompatibility with certain active compounds or environments [5,6]. These challenges have driven the development of alternative gel systems with enhanced functionalities.

**Figure 1:**
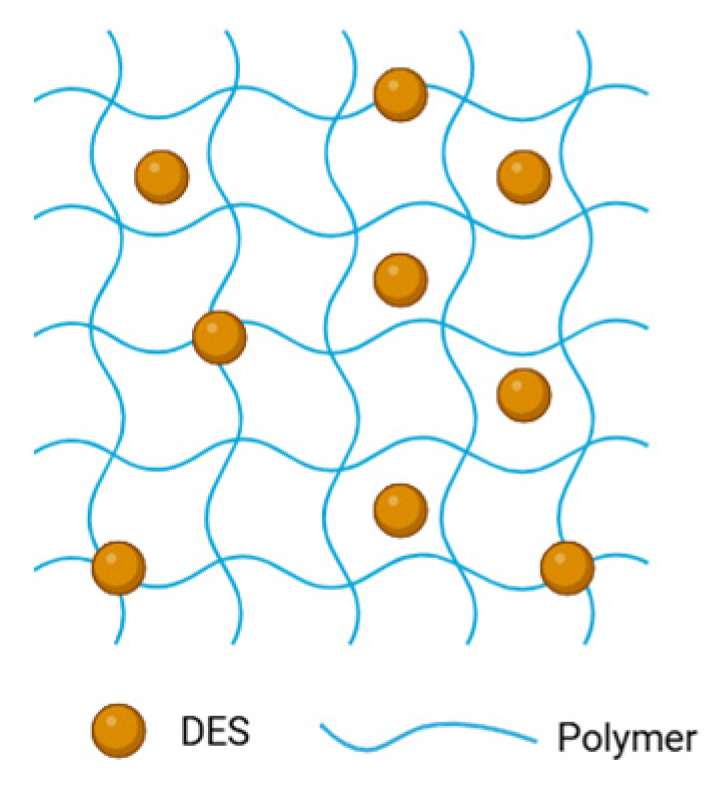
Conceptual illustration of an eutectogel 3D network (created with BioRender.com).

Eutectogels have recently emerged as a promising class of soft materials that combine the properties of eutectic solvents with the structural integrity of polymeric gel networks. Figure 1 illustrates the 3D network of an eutectogel, in which it is possible to observe the presence of the DESs within the polymeric network. Defined as gels in which the liquid phase consists of deep eutectic systems (DESs) or natural deep eutectic systems (NADESs), eutectogels exhibit unique physicochemical characteristics inherited from their eutectic components, typically hydrogen bond donors and acceptors that form a stable, low-melting mixture. The resulting gels benefit from the intrinsic advantages of DESs, including low volatility, biodegradability, high solubilization capacity for a wide range of compounds, and potential biocompatibility.

**Figure 2:**
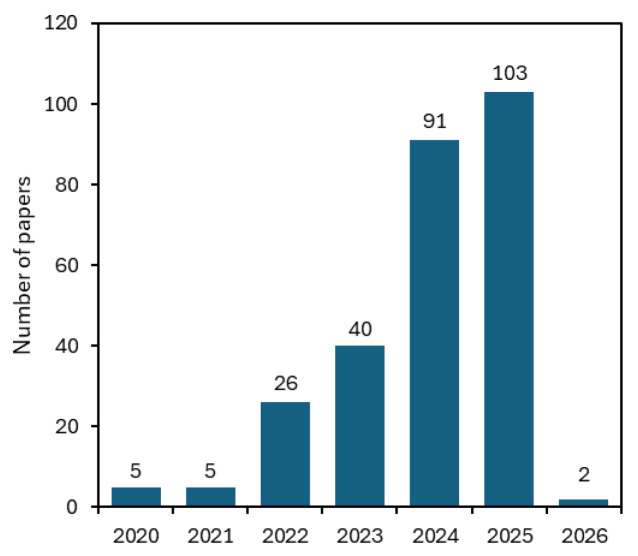
Number of publications concerning eutectogels, since 2020. (Search from Web of Knowledge, conducted on 5 November 2025, with the keyword “eutectogels”).

While the broader field of gel research (e.g., hydrogels) has been established for decades, gel-like materials formed from DESs emerged in the early 2000s. However, the term “eutectogel” has only been reported for the first time in 2020. Since then and especially following a landmark review published by Wang et al. in 2021 [7], the number of studies on eutectogels had a sharp increase after 2022, reflecting a shift from proof-of-concept studies toward application-driven research (Figure 2). This trend highlights eutectogels as a rapidly expanding area at the intersection of gel science and green solvent engineering.

Many reported eutectogels show outstanding thermal and chemical stability, ionic conductivity, self-healing capabilities, and customizable rheological properties [4,8,9]. However, this is not a universal characteristic since it is highly dependent on the network type, the crosslinking mechanism, water content, and even the composition of the DESs [10,11,12].

Eutectogels’ design versatility, through the selection of eutectic components and gel-forming polymers, enables fine-tuning of their mechanical strength, responsiveness to stimuli, and compatibility with specific applications [13,14,15]. These attributes make eutectogels attractive candidates for use in areas such as drug delivery, energy storage, sensors, electrochemical devices, and environmentally friendly materials.

This review provides an extensive overview of the advances in the field of eutectogels, focusing on their design strategies, physicochemical properties, and applications. By outlining the current state of the art and identifying future challenges, this review seeks to inform and inspire innovation in the field of eutectogels.

## 2. Eutectogels

### 2.1. Deep Eutectic Systems

DESs were first described by Abbott and coworkers in 2001 [16]. In that study, different quaternary ammonium salts were mixed and heated with ZnCl_2_ and interestingly, when choline chloride (ChCl) was used, the melting point of such mixture was 23–25 °C, i.e., liquid at room temperature. Later, other liquids were prepared by the combination of salts and hydrogen bond donors (HBDs) and finally the term “deep eutectic systems” was established [17]. DESs are mixtures that are liquid close to their eutectic composition, i.e., the molar ratio of the components which gives the lowest melting point. Usually, they are composed of hydrogen bond donors (HBDs) and hydrogen bond acceptors (HBAs). The common HBAs include quaternary ammonium salts, particularly ChCl, while HBDs can range from amides and carboxylic acids to polyols and sugars. The unique physicochemical properties of DESs, such as low volatility, biodegradability, thermal stability, and tunable polarity, make them attractive alternatives to traditional organic solvents [18,19,20]. Due to their ease of preparation, low cost, and customizable nature, DESs have found widespread applications in green chemistry, extraction processes, electrochemistry, catalysis, and pharmaceutical formulations.

Moreover, DESs can also serve as functional media for the preparation of eutectogels. Their high viscosity and strong hydrogen bonding capability contribute to the physical stability and unique mechanical properties of eutectogels [8]. These gels combine the tunable physicochemical characteristics of DESs with the structural versatility of polymers, making them ideal for applications in drug delivery, sensors, electrochemical devices, and soft actuators.

### 2.2. Types of Eutectogels

Eutectogels can be broadly grouped into polymeric [13,21,22], supramolecular [23,24,25] or biopolymer-based systems, which are eutectogels that are composed of natural macromolecules such as chitosan, alginate, cellulose, starch, gelatine, agarose, dextran, or silk fibroin as the primary network-forming agents.

Eutectogels can be further divided according to their crosslinking mechanism (chemically vs. physically crosslinked) [26,27,28] and structural arrangement (homogeneous vs. heterogeneous) [29]. The microstructural organization and crosslinking strategy further diversify the eutectogel properties. Physically crosslinked eutectogels, stabilized by hydrogen bonding, electrostatic forces, or supramolecular interactions, exhibit features such as self-healing, injectability, and recyclability, whereas chemically crosslinked eutectogels offer greater long-term stability, solvent retention, and mechanical resistance. Hybrid strategies that combine both reversible and permanent crosslinking are emerging as promising solutions to balance adaptability with durability. Likewise, homogeneous networks, with uniform ionic distribution, generally yield predictable electrochemical behavior, while heterogeneous systems, often obtained through phase separation or templating, allow spatial compartmentalization of functions such as conductivity, swelling, or stiffness. Therefore, the choice of gel type largely depends on the targeted property, whether it is mechanical robustness, biocompatibility, environmental stability, or conductivity [30].

From a functional standpoint, eutectogels may also be categorized according to the role of DESs. They can be classified as solvent-type eutectogels, in which DESs act exclusively as the solvent, or as monomer-derived eutectogels, where DES components serve simultaneously as solvent and reactive precursors [28]. In most reported systems, DESs primarily function as solvents, being immobilized within a polymeric or supramolecular network. In this configuration, the DES phase provides ionic conductivity, anti-freezing ability, and solvation capacity, while the surrounding network contributes structural integrity and mechanical robustness.

More recently, research has moved toward the design of eutectogels in which DES constituents are not merely passive fillers but participate directly in polymerization or crosslinking reactions. In these monomer-derived systems, DES molecules are covalently integrated into the gel backbone, thereby improving stability, reducing leaching, and enabling the incorporation of functional groups from DESs into the polymer network. This strategy enhances durability, mechanical performance, and multifunctionality, opening new opportunities for flexible electronics, bio-interfaces, and advanced biomedical devices [31].

Taken together, the classification of eutectogels thus requires consideration of (i) the role of DESs (solvent vs. monomer), (ii) the type of network (polymeric, supramolecular, biopolymeric), (iii) the crosslinking mechanism (physical vs. chemical), and (iv) the structural arrangement (homogeneous vs. heterogeneous). Each dimension contributes uniquely to the final material properties and application potential. Accordingly, Table 1 summarizes the different categories of eutectogels, highlighting their key features, advantages, and limitations, as well as their most relevant application scenarios.

### 2.3. Key Properties of Eutectogels

The performance of eutectogels is determined not only by their composition but also by their structural organization and the role of DESs within the network. Several key properties define their functionality and guide their application potential.

#### 2.3.1. Mechanical Properties

The mechanical performance of eutectogels is primarily dictated by their network architecture and crosslinking strategy. Chemically crosslinked networks generally provide higher stiffness, structural stability, and load-bearing capacity, making them suitable for applications requiring long-term durability. In contrast, physically crosslinked gels, stabilized through reversible interactions such as hydrogen bonding, ionic interactions, or host–guest complexes, exhibit enhanced flexibility, injectability, and self-healing behavior [32,33]. These contrasting behaviors arise from fundamental differences in bond lifetime and energy dissipation. Covalent crosslinks possess high bond dissociation energies and long lifetimes, creating a permanent network that resists deformation and prevents chain rearrangement, thereby enhancing mechanical robustness and creep resistance. In contrast, physically crosslinked networks are stabilized by reversible interactions with shorter lifetimes, allowing the network to reorganize under stress, dissipate energy efficiently, and rapidly reform once the stress is removed [34]. DESs incorporated within these networks serve as effective plasticizers, improving stretchability and deformability; however, excessive DES content can compromise the gel’s mechanical integrity by weakening the polymer network [35]. Consequently, the mechanical properties of eutectogels can be finely tuned through the careful selection of polymer type, crosslinking density, and DES composition, enabling the design of materials that balance toughness, elasticity, and adaptability for applications ranging from soft electronics to biomedical scaffolds.

**Table 1:** Types of eutectogels, key features, advantages, and differences and similarities.

Category	Subtype/ Classification	Role of DESs	Key Features/ Properties	Differences and Similarities	Reference
Network type	Polymeric Supramolecular Biopolymer-based	Solvent only or solvent + monomer	Mechanical robustness, conductivity, biocompatibility, environmental stability	Polymeric: tunable mechanics; Supramolecular: reversible, dynamic networks; Biopolymer: biocompatible, biodegradable	[21,22,25,36,37]
Crosslinking	Chemically vs. Physically crosslinked	-	Chemical: covalent, stronger; Physical: non-covalent, reversible	Chemically and physically crosslinked gels can both provide flexibility; chemical more stable, physical more dynamic	[26,38,39]
By structure	Homogeneous vs. Heterogeneous	-	Homogeneous: uniform properties; Heterogeneous: phase-separated, multifunctional	Both can provide ionic conductivity; heterogeneous may have superior mechanical strength or multifunctionality but are more unstable	[31]
By DES role	DESs as solvent only	Passive, provides conductivity, anti-freezing, solvation	Network provides structure; DES acts as filler	Good flexibility and tunable ionic transport; DESs not covalently bound	[7,40,41]
	DESs as solvent + monomer	Active, participates in polymerization or crosslinking	Covalent incorporation of DESs; improved stability, mechanical integrity, multifunctionality	Enhanced durability and chemical stability; multifunctional; less flexibility than solvent-only gels	[42,43,44]

In the literature, numerous studies have demonstrated the successful design of eutectogels with remarkable mechanical properties. For instance, Jiang et al. [32] developed eutectogels with a high Young’s modulus of 103.1 MPa and fracture energy of 98.7 kJ m^−2^, achieved through a solvent exchange process that optimized crystalline domain formation. Similarly, Lu et al. [33] reported eutectogels exhibiting a tensile strength of 8.04 MPa and elongation at break of 620.5%, achieved via a physically entangled network in deep eutectic solvents. Similarly, Gu et al. [29] constructed organic–inorganic hybrid eutectogels by embedding alkaline calcium bentonite (ACBT) within a PAM/PVP matrix. The resulting hybrid network achieved high tensile strength (1.09 MPa), elongation (1411%), and adhesion (113.7 kPa), demonstrating the effectiveness of inorganic–polymer synergy in enhancing mechanical robustness and multifunctionality.

These examples underscore the versatility and potential of eutectogels in various technological and biomedical applications.

#### 2.3.2. Thermal Stability and Anti-Freezing Behavior

A distinctive advantage of eutectogels is their intrinsic anti-freezing capability, arising from the freezing-point depression of DESs [45,46]. This enables them to retain ionic conductivity and flexibility under sub-zero temperatures, making them ideal for outdoor energy and cryogenic biomedical applications. For example, Heydari et al. [47] developed a gelatine-based conductive eutectogel from glycidyl methacrylate-modified gelatine and a polymerizable DES (citric acid:dimethyl diallyl ammonium chloride, 1:2). The material exhibited outstanding stretchability (~1789%), tensile strength (0.25 MPa), conductivity (13.75 mS cm^−1^), and sensitivity (gauge factor = 2.71), along with rapid self-healing, strong adhesion, anti-freezing and anti-drying stability, and antibacterial activity. It maintained flexibility and sensing performance across extreme temperatures, effectively detecting fine human motions such as swallowing and speaking, underscoring its potential for wearable and biomedical electronics.

Similarly, Hu et al. [48] reported a dual-bioinspired eutectogel combining proline: sorbitol-based NADESs and hydroxylated carbon nanotubes within a calcium alginate network. Mimicking the anti-freeze fluids of cold-tolerant organisms and the photothermal absorption of poikilotherms, this design yielded eutectogels with excellent mechanical strength, flexibility, and photothermal conversion efficiency (~49%). The optimized formulation exhibited superior anti-frosting, de-icing, and moisture regulation, maintaining stable performance under sub-zero conditions and efficiently converting solar energy into heat.

#### 2.3.3. Ionic Conductivity and Electrochemical Properties

DESs contain ionic or hydrogen-bonding species [49,50], therefore they endow eutectogels with intrinsic ionic conductivity, enabling efficient ion transport through the polymer network. Conductivity can be tuned via DES composition, concentration, and network density, making eutectogels ideal for solid-state electrolytes, electrochemical sensors, and bioelectronic devices. Yang et al. [51] developed a highly conductive, self-healing, and temperature-tolerant zwitterionic eutectogel for triboelectric nanogenerators, prepared by photopolymerizing DMAPS and acrylic acid in an allyltrimethylammonium chloride and ethylene glycol-based DES. The material achieved a record ionic conductivity of 15.7 mS cm^−1^ and maintained stable performance from −80 °C to 100 °C. Integrated with patterned polydimethylsiloxane, it generated a maximum power density of 112 mW m^−2^ and functioned as a self-powered motion sensor. Likewise, Joos et al. [9] introduced DESs–silica eutectogels as solid composite electrolytes for lithium-based batteries. These materials combined the processability of DESs with the rigidity of silica, achieving high ionic conductivity (1.46 mS cm^−1^), broad electrochemical stability (1.1–4.8 V vs. Li^+^/Li), and excellent cycling performance (>99% Coulombic efficiency) in Li/ETG/LiFePO_4_ cells.

It is worth noting that the ionic conductivities reported for eutectogels (typically in the 1–20 mS cm^−1^ range) are comparable or even enhanced to those of many state of the art hydrogels and ionogels (ionic liquid-based gels), although the direct quantitative benchmarking across these distinct material classes may be complicated due to differences in the composition, solvent content, and measurement conditions [8,52,53].

#### 2.3.4. Solvent Retention and Swelling

Eutectogel stability relies on immobilizing DESs while minimizing leaching, a balance that is governed by the polymer, DES interactions, and the network architecture. On the one hand, chemically crosslinked networks effectively immobilize the DESs but have limited swelling and flexibility. On the other hand, physically crosslinked gels have higher swelling degrees but leach DESs over time. Hybrid networks, combining covalent and reversible supramolecular interactions, along with hydrogen-bond-matched polymers or nanofillers, enhance DES retention without sacrificing adaptability. Optimizing the immobilization of DESs in polymeric matrixes ensures consistent properties, such as mechanical strength and safety, making eutectogels suitable for wearable sensors, solid-state batteries, and flexible ionic devices.

An example is the work of Lu et al. [54], who developed a hydrophobic DES-based eutectogel with exceptional long-term humidity resistance and multifunctional sensing performance. Synthesized via rapid photopolymerization of acrylic acid in a tetrabutylammonium bromide/*n*-octanol, the gel forms a dual-barrier structure that limits water permeation while maintaining ionic conductivity. The eutectogel exhibited minimal water absorption (2.8% at 30 °C, 90% RH for 30 days), retained 99.3% toughness after one year, and combined high stretchability (828%), tensile strength (2.43 MPa), adhesion (up to 922 kPa), self-healing (~85%), and thermal stability (−20 °C to 110 °C). It also displayed complete antibacterial activity and stable sensing performance under humidity and temperature fluctuations, demonstrating the practical potential of optimized eutectogel designs for next-generation wearable and environmental sensors.

However, quantitative comparisons across eutectogel systems remain limited since solvent retention, swelling ratios, and diffusion coefficients strongly depend on DES type, hydrogen-bonding strength, polymer chemistry, crosslinking density, and environmental conditions (temperature, humidity, and water activity). Extracting universal numerical ranges is difficult since the reported values are widely varied, with swelling ratios ranging from negligible (<5%), in tightly crosslinked hydrophobic DESs [10], to more than 30%, in physically crosslinked hydrophilic eutectogels [55,56].

#### 2.3.5. Self-Healing and Stimuli Responsiveness

Eutectogels formed through reversible interactions, such as hydrogen bonding, electrostatic attraction, or host–guest inclusion, often display autonomous self-healing and responsiveness to external stimuli including pH, temperature, light or electrical fields. These dynamic features are highly desirable for actuators, smart coatings, wearable sensors, and adaptive biomedical scaffolds.

Self-healing eutectogels are particularly relevant because they combine mechanical strength with functional recovery, addressing the durability limitations of conventional soft materials. A brief search in Web of Knowledge using the term “self-healing eutectogels” retrieved approximately 150 documents, about 87 of which were published in the past three years, underscoring the growing research activity on eutectogels with self-healing properties.

The incorporation of DESs within polymeric or supramolecular networks enables dynamic hydrogen-bonding and ionic interactions that spontaneously re-establish structural integrity after damage, restoring both mechanical strength and ionic conductivity. This reversibility supports long-term stability and performance in flexible electronics, energy devices, and biomedical applications, aligning with principles of green and sustainable material design [8,22,57]. Nevertheless, the review published by Ding et al. [30] discusses that eutectogels still have deficiencies in tensile properties, self-healing ability, and conductive performance, suggesting the improvement of such properties remains one of the central research challenges. In the same perspective, stimuli-responsive eutectogels can also modulate their structure and properties under environmental changes, providing tunable and controllable functionality [58]. The presence of DESs allows adjustable ionic mobility and hydrogen-bond rearrangement, enabling precise control of swelling, mechanical strength, and molecular release. Their compositional versatility allows the design of single or multi-stimuli-responsive systems, positioning these materials as adaptable and multifunctional platforms for advanced sensing, actuation, and therapeutic applications [24,59].

In the same manner stimuli-responsive eutectogels also have some limitations. Their mechanical robustness is often inadequate, with many systems exhibiting low strength, slow recovery or poor fatigue resistance under repeated stimulation [60,61]. Responsiveness itself can be restricted by narrow operating windows, slow reaction times, and limited reversibility, while the intrinsic properties of DESs, such as hygroscopicity, viscosity changes, and possible component leaching, introduce additional challenges for stability and reproducibility [7,35,54].

In the literature, numerous examples have demonstrated the successful design of self-healing [62,63,64,65,66,67] and stimuli-responsive eutectogels [14,68,69,70,71] with remarkable mechanical robustness, ionic conductivity, and environmental stability, reinforcing their potential as next-generation smart materials for diverse technological and biomedical applications.

#### 2.3.6. Biocompatibility and Biodegradability

Eutectogels produced with natural polymers are particularly interesting for biomedical applications considering their renewable origin, intrinsic biocompatibility, and inherent biodegradability. NADESs have been widely described for their biocompatibility [72,73,74] and their incorporation into gels can help in the modulation of biological interactions, (i.e., by influencing cell adhesion and proliferation), as well as in the optimization of drug release profiles. Nevertheless, the cytotoxicity of eutectogels depends on multiple factors such as the DES components and molar ratios and the concentration within the polymer matrix [75]. Therefore, a careful selection of both DES and polymer constituents should be made to ensure safe and effective performance for its desired applications. Optimizing the composition and network architecture of biopolymer-based eutectogels allows us to achieve the needed mechanical properties, biodegradability, and biological compatibility, enabling their translation into functional biomedical devices and therapies. Ideally, DESs should be composed of molecules already described as biocompatible, namely, primary metabolites such as polyols (e.g., glycerol, sorbitol, xylitol, etc.), sugars (e.g., glucose, trehalose, fructose, etc.), organic acids (e.g., malic acid, oxalic acid, etc.) or choline derivatives (e.g., betaine, acetyl choline), which the human body can easily recognize and metabolize. Nevertheless, each combination must be evaluated independently. For instance, despite being prepared from naturally occurring molecules, acidic DESs may present a certain level of cytotoxicity, which results in the shift in cell media pH and consequent cell death [24,76].

Several studies have demonstrated the successful design of biopolymer-based eutectogels with remarkable biocompatibility [8,77,78,79,80,81]. For example, Xia et al. evaluated the toxicity of an eutectogel composed of xanthan gum, ChCl, and xylitol (Xyl), on HepG2 cell line and concluded that the formulation was non-toxic in the conditions tested [77]. Other choline-based eutectogels also exhibited non-toxic profiles against human gingival fibroblasts [79], human lung fibroblasts (MCR-5) [78,81], human keratinocytes (HaCaT) [78], and human umbilical vascular endothelial cells [82]. Moreover, the hemocompatibility of eutectogels has also been demonstrated [83].

Concerning the biodegradability of eutectogels, there are fewer studies focusing on this subject. One example found in the literature is the one reported by Mercadal et al. who evaluated the degradability of ethaline and cellulose nanocrystals eutectogels and observed that 6 days in controlled composting conditions led to 60% to 90% weight loss, indicating their biodegradable profile [81]. These findings highlight the potential of eutectogels to serve as environmentally sustainable soft materials, particularly when formulated with naturally derived polymers or nanomaterials. Nevertheless, biodegradability is also dependent on DES composition and polymeric network structure, and studies on long-term environmental impact, metabolite formation, and degradation under diverse conditions are still limited. Further research into these aspects is essential to validate their suitability for green electronics, biomedical devices, and transient material applications.

Overall, for biotechnological applications, the most critical characteristics of eutectogels are their biocompatibility, ability to maintain biological activity, tunable permeability for controlled transport of biomolecules, and mechanical properties that mimic soft tissues.

## 3. Applications of Eutectogels in Biotechnology

**Figure 3:**
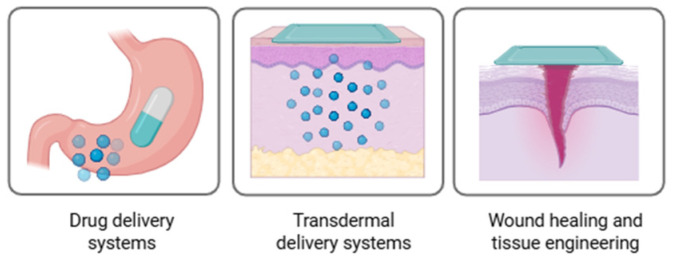
Conceptual illustration of the main applications of eutectogels in biotechnology (created with BioRender.com).

As previously described, eutectogels find applications in multiple fields such as energy storage and electronics (including electrochemical devices and electrolytes for batteries and supercapacitors), wearable and flexible devices (strain and pressure sensors and underwater electronics), adhesives and coatings, separation and catalysis (chromatographic separation materials and catalyst supports), and biomedical and biopharmaceutical. In this section we will focus on the advances on the biotechnological applications of eutectogels. From the research conducted, eutectogels in this category can be subdivided into three major groups: drug delivery systems (DDSs), transdermal delivery systems (TDDSs), and eutectogels for wound healing and tissue engineering, as conceptually illustrated in Figure 3.

### 3.1. Drug Delivery Systems

Given the ability of DESs to enhance both the solubility and stability of active pharmaceutical ingredients (APIs), eutectogels have recently emerged as promising DDSs. These systems are designed to enable the safe, effective, and controlled administration of therapeutic agents. The DES, gelator, gelation method, and main properties of the examples herein described are summarized in Table 2.

In general, for drug delivery applications, chemically crosslinked polymer networks are usually the most efficient retaining DESs, while providing stable, predictable diffusion pathways for controlled drug release.

The first report of the use of an eutectogel based on natural polysaccharides with potential to be used as a DDS was published in 2020 by Xia et al. [77]. The authors used a DES composed of ChCl and xylitol, containing 20% water, that enhanced the solubility of quercetin by 10,000 times compared to its solubility in pure water. Then, using xanthan gum as gelator, they developed a material that, in comparison to the corresponding hydrogel, showed improved viscoelastic properties, higher thermal stability, and a more defined shear behavior. Furthermore, no toxicity was observed towards HepG2 cells, revealing a high biocompatibility.

Addressing the growing global concern regarding antimicrobial resistance, Shaw et al. [84] prepared cellulose-based eutectogels incorporating multiple antimicrobial agents, including nanomaterial-based and conventional pharmaceutical compounds. The antimicrobial activity of these eutectogels was evaluated against pathogenic species commonly associated with infected wounds, including *Staphylococcus aureus* (MRSA), *Pseudomonas aeruginosa*, and *Candida albicans*. The formulations achieved more than 97% reduction in viable cells for all tested microorganisms, confirming their potent antimicrobial efficacy.

In the field of oncological drug delivery, Parsana et al. [70] developed a self-healing, injectable, and biocompatible eutectogel capable of polymerizing in response to physiological stimuli such as pH and temperature. Using biocompatible precursors such as ChCl, glycerol, and poly(vinyl alcohol), the researchers obtained a gel with high mechanical strength (>7.3% strain) and enhanced cytocompatibility, as demonstrated in assays with red blood cells and human skin cells (HaCaT). This system enabled sustained release of anticancer drugs (5-fluorouracil, 5-FU) under physiological conditions. Furthermore, the toxicity of 5-FU-loaded eutectogels was also tested towards MCF-7 and HeLa cells, showing promising selectivity results as anticancer agents. The same group also described a ChCl-based eutectogel incorporating a metal–organic framework (MOF), ZIF-8. The resulting material was injectable, adhesive, and self-healing, with an exceptional curcumin loading capacity (>70,000-fold higher than in water) and a controlled release profile under acidic conditions. Furthermore, the eutectogel exhibited antimicrobial and antioxidant activities, highlighting its potential for diverse pharmaceutical applications, including wound healing and localized drug delivery [83].

Albertini et al. [85] proposed a novel approach for the pediatric administration of benznidazole (BNZ) through the development of an eutectogel composed of ChCl-based NADESs and xanthan gum. The researchers were able to prepare eutectogels loaded with 5 mg/mL of BNZ. After oral administration in rats, they observed that the drug bioavailability increased by 2.6-fold compared to the solid form of the drug, also being detected in the rats’ cerebrospinal fluid. Designed for oral application, this system demonstrated favorable rheological behavior and excellent biocompatibility, making it a suitable platform for pediatric formulations.

In another study focused on oral mucosal delivery, Anuta et al. [86] developed an eutectogel based on xanthan gum (XTG), hyaluronic acid (HA), and the DES ChCl:sorbitol:glycerol (2:1:1), incorporating 2.5% ibuprofen as a model drug. The eutectogel exhibited shear-thinning behavior, high mucoadhesiveness, and a sustained release of ibuprofen over 24 h. The authors studied the ex vivo mucosal residence time, which revealed that the formulations differed substantially in performance, with the DESs playing a key enhancing role. The base formulation, XTG-HA gel (without DESs), showed a moderate residence time of about 72 min, while the incorporation of DESs significantly increased mucosal retention to *circa* 183 min, indicating that the DESs markedly reinforce polymer–mucus interactions, mostly by improving hydration, promoting polymer entanglement, and strengthening adhesion to mucosal glycoproteins. This formulation also displayed anti-inflammatory and antibacterial properties, supporting its applicability in oral cavity treatments.

In a different strategy, Meneses et al. [87] designed an enzymatically crosslinked eutectogel intended for injectable applications. Using an NADES composed of betaine and glycerol (1:2) combined with gelatine, the authors obtained a material with shear-thinning behavior and enhanced rheological performance compared to conventional hydrogels, remaining stable for at least one year. The eutectogel facilitated the sustained release of the anti-inflammatory drug ketoprofen over a 10-day period. Similarly, Cheng et al. [88] developed injectable eutectogels for the treatment of chronic periodontitis using ChCl, lysozyme fibers, and gallic acid. The obtained material exhibited shear-thinning and adhesive behavior, enhancing pharmacological performance. The gels exhibited near-complete bactericidal activity and modulated immune responses (reducing CD86^+^ cells and increasing CD206^+^ cells) and effectively inhibited alveolar bone loss and local inflammation in a rat model of chronic periodontitis. These results support the evidence that self-assembled eutectogels can enhance therapeutic efficacy against inflammatory diseases by promoting bacterial control, reactive oxygen species scavenging, and macrophage regulation through improved delivery of small-molecule drugs.

To enhance the solubility of an antifungal drug, Chachad et al. [89] developed an adhesive eutectogel incorporating two polymers (sodium alginate and hydroxypropyl methylcellulose) within a ChCl:propylene glycol system. The gel demonstrated favorable viscosity, pH, and spreadability. Additionally, the use of NADESs in this formulation significantly increased the drug’s solubility, while providing a sustained release profile over 12 h (93.85 ± 2.81%), and antifungal activity comparable to a marketed 1% formulation. Owing to its adhesive properties and controlled-release behavior, this eutectogel represents a promising platform for the topical delivery of antifungal therapeutics.

More recently, the formation of eutectogels without the need for an external carrier has been proposed. Yuan et al. [90] described the preparation of a therapeutic deep eutectic solvent (THEDES) composed of oxymatrine and lauric acid (3:7), which spontaneously formed a gel upon the addition of water. Although oxymatrine is already known for its therapeutic effects, including antiviral, anti-inflammatory, and antitumor properties, the authors still incorporated curcumin to further improve the bioactivity of the gel. Scanning electron microscopy (SEM) analysis revealed a well-defined 3D network, attributed to hydrophobic interactions among lauric acid molecules. The THEDES formulation exhibited antibacterial activity against *Propionibacterium acnes*. Considering all these properties, the authors suggested that this simple and carrier-free eutectogel preparation method holds great potential for broad biomedical applications.

An example of a real-world application of eutectogels is described in EP4543465A1 submitted by RMIT University and Melbourne Institute of Technology [91]. The patent discloses an antimicrobial eutectogel comprising a DES, a polymeric gelling agent (e.g., a cellulose-derived or polysaccharide polymer), and an antimicrobial agent (such as metallic nanoparticles or conventional antibiotics). This eutectogel is capable of sustaining a moist environment and enables the delivery of both water-soluble and water-insoluble antimicrobials over extended periods. These properties illustrate how their unique solvent retention and solvation properties can translate into practical advantages over conventional water-based hydrogels for biomedical applications (e.g., topical antimicrobial dressings).

**Table 2:** Summary table with DES and gelator used, gelation method, and main properties of the eutectogels reported as drug delivery systems.

DES	Gelator	Gelation Method	Properties	References
ChCl:xylitol	Xanthan gum	Temperature induced self-assembly	Shear-thinning behavior; Improved viscoelastic properties; High thermal stability; Biocompatibility	[77]
ChCl:glycerol (1:2)	Cellulose	Self-assembly	High adhesion; Flexible nature; Shear-thinning behavior	[84]
ChCl:Imidazole (3:7); Tetrabutylammonium bromide:Imidazole (1:2); Tetrabutylphosphonium bromide:Imidazole (1:1)	Polyvinyl alcohol (PVA)	Self-assembly	Strong mechanical strength (>7.3% strain); Swelling; Self-healing; Biocompatibility; Stimuli responsive	[70]
ChCl:Fructose (2:1) ChCl:Glucose (2:1)	ZIF-8 Sodium alginate	Temperature induced intermolecular non-covalent interactions	Injectability; Adhesion; Hemocompatibility	[83]
ChCl-based	Xanthan gum	Temperature induced self-assembly	Viscoelastic behavior; Higher loading of drug; High bioavailability	[85]
Betaine:glycerol (1:2)	Gelatine-phenol conjugate	Enzymatic crosslinking	Shear-thinning behavior; Resistant material; Biocompatibility	[87]
ChCl:mannose	Lysozyme fibers and gallic acid	Self-assembly through hydrogen bonding and hydrophilic/hydrophobic interaction	Shear-thinning behavior; Adhesive; Antimicrobial; Anti-inflammatory	[88]
ChCl:sorbitol:glycerol (2:1:1)	Xanthan gum Hyaluronic acid	Temperature induced physical interactions (hydrogen bonding, polymer entanglement, and electrostatic interactions)	Shear-thinning; High mucoadhesiveness	[86]
Oxymatrine:lauric acid (3:7)	-	Self-aggregation in water	Strong shear-thinning behavior; Strong mechanical properties; Improvement of curcumin solubility and stability; Antibacterial capacity	[90]
ChCl:propylene glycol (1:2)	Hydroxypropyl methylcellulose K15 (HPMC K15) Sodium alginate	Self-assembly after hydration	Good viscosity and spreadability; High drug loading; Sustained release profile	[89]

### 3.2. Transdermal Delivery Systems

Although transdermal drug delivery systems (TDDSs) fall into the DDSs category, TDDSs are a specific class of delivery systems which enables the transport of active pharmaceutical ingredients (APIs) across the skin and directly into the bloodstream. These systems offer several advantages, including the avoidance of first-pass metabolism, non-invasive administration, and controlled drug release, which together maintain consistent concentration of the drugs in plasma. However, conventional hydrogels used in TDDSs are limited by poor adhesion, low cohesive strength, and instability outside narrow temperature ranges [92]. To address these shortcomings, eutectogels have been investigated as advanced materials for transdermal applications. Table 3 summarizes the conditions used for the preparation of transdermal eutectogels as well as their main properties.

In 2024, Picchio et al. [78] were the first to report a protein–elastomer therapeutic eutectogel composed of choline:geranic acid (CAGE, 1:2) and gelatine to promote the dermal penetration of hydrophobic and hydrophilic molecules. Although CAGE has an ionic nature, it was able to stabilize the secondary structure of gelatin, which afforded materials with long stress–relaxation times (60% decay after 500 s) similar to those of chemically crosslinked networks. Leveraging the distinctive properties of CAGE, namely, its intrinsic antimicrobial activity and skin permeation enhancement, the authors developed an eutectogel that, when combined with tannic acid (TA), exhibited high strength and flexibility, mechanical characteristics rarely achieved with conventional biopolymeric matrices.

Zhang et al. [92] developed a calcium ion-coordinated *p*-hydroxyphenyl methacrylate (HP-Ca^2+^) eutectogel using ChCl:ethylene glycol (1:2) as solvent. The eutectogel exhibited superior physicochemical and mechanical properties compared to conventional hydrogels composed of polyacrylic acid (PAA), namely, strong adhesion (>30 kPa), high mechanical strength (>300 kPa), stretchability (>1600%), and thermal tolerance (−25 to 60 °C), enabling sustained and controlled drug release. Using model drugs (flurbiprofen and sodium diclofenac), the eutectogels loaded higher amounts of each drug and the obtained release profile was comparable to those of PAA hydrogels. Moreover, both in vitro and in vivo assessments confirmed its biosafety, underscoring its potential as a next-generation transdermal carrier.

Yang et al. [93] reported the use of a THEDES composed of matrine and lauric acid (3:7) for the development of a transdermal patch for the treatment of acne infections. In this work, the THEDES works as a gelator itself, since it self-assembles into eutectogel in the presence of water. The eutectogel formation was confirmed by its rheological analysis and through scanning electron microscope (SEM) analysis. In addition, the THEDES allowed the solubilization and stabilization of curcumin and the resulting eutectogel exhibited antioxidant and antimicrobial activity against *P. acnes*. Once again, the results highlight the relevance of the DES presence in the final gel.

Atenolol, a β-blocker commonly used to treat hypertension, was used by Cassano et al. [55] to develop a new TDDS comprising the ChCl:propylene glycol DES. Solubility and thermodynamic analyses confirmed strong hydrogen-bond interactions between atenolol and the DES. The eutectogel was composed of gelatine or Tego^®^ Carbomer 140 and their rheological behavior, swelling ability, and drug permeation using Franz diffusion cells were studied. Superior drug release profiles were observed when compared to equivalent hydrogels, achieving approximately 86% release for Carbomer-based and 51% for gelatin-based formulations, whereas traditional hydrogels released only about 27% and 35%, respectively. Overall, these findings demonstrate that eutectogels offer a promising and more efficient alternative to conventional hydrogels for enhancing the topical delivery of atenolol.

Li et al. [94] proposed an eutectogel-based TDDS for the treatment of psoriasis, a chronic inflammatory skin disorder. The eutectogel was prepared using chitosan and the DES (2-hydroxypropyl)-β-cyclodextrin:levullinic acid (1:7). This formulation demonstrated high skin adhesiveness and served as an effective carrier for resveratrol, an API with anti-psoriatic potential. The DES not only functioned as a dispersion medium for chitosan, due to its acidity, but also acted as a crosslinking and plasticizing agent, enhancing mechanical integrity while simplifying the preparation process. Additionally, it improved API solubility and prolonged stability, confirming its suitability for long-term therapeutic applications. In a psoriasis-induced mouse model, it significantly reduced symptoms, suppressing the keratinocyte overproliferation, and modulated the cytokines IL-23/IL-17, which are related to inflammatory pathways, outperforming commercial calcipotriol ointment. Overall, this DES-enhanced eutectogel shows strong potential as a topical delivery platform for psoriasis treatment and broadens the use of environmentally friendly polysaccharide-based medical materials.

Fatahi et al. [95] developed an eutectogel using the natural polysaccharide bassorin as the polymer matrix for the transdermal delivery of dextromethorphan (DXM). Incorporation of the DES malic acid:ChCl (1:2) improved both drug solubility and skin permeation. In vivo studies demonstrated the eutectogels’ anti-inflammatory effects, and its therapeutic efficacy, namely, the paw swelling rate and reduction of TNF-α levels. As important to note, these results were comparable to oral DXM formulations but without associated systemic side effects. Additional histological studies showed that the rats’ knee joints had few abnormalities.

Microneedles (MNs) emerged as an innovative and minimally invasive transdermal drug-delivery technology capable of overcoming the inherent barriers of the skin, particularly the stratum corneum. By incorporating arrays of micro-sized projections that painlessly penetrate the superficial skin layers, microneedles enable efficient delivery of therapeutic molecules that would otherwise exhibit poor permeation through conventional topical routes. Their unique design allows for controlled and localized administration of a wide range of agents, including small molecules, peptides, proteins, and vaccines, while minimizing systemic side effects and improving patient compliance [96,97].

**Figure 4:**
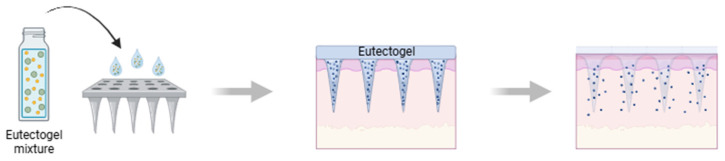
Schematic illustration of the development of microneedle patches for transdermal drug delivery, using eutectogels (created with BioRender.com).

Recently, the integration of eutectogels into microneedle design (Figure 4) has opened new possibilities for enhancing both mechanical performance and drug-delivery efficiency. Owing to their unique combination of flexibility, high drug-loading capacity, and tunable physicochemical properties, eutectogels provide a robust yet adaptable matrix for MN fabrication. Their inherent ability to solubilize poorly soluble drugs, promote controlled or stimuli-responsive release, and improve skin interfacing makes them particularly attractive for next-generation transdermal systems. As a result, eutectogel-based MNs represent a promising advancement that unites the structural advantages of microneedle technology with the functional versatility of deep eutectic solvent-derived materials [96,98].

Liu et al. have extensively studied the development of eutectogel-based MNs. For instance, they [99] developed MN patches composed entirely of polymerizable DESs for both the microneedle and backing layers. The patches were formulated with ChCl, 2-hydroxyethyl methacrylate (HEMA), and itaconic acid (IA) for the MNs and glycerol for the backing layer. The use of a polymerizable DES enabled rapid photopolymerization and excellent mechanical strength for effective skin penetration. This one-step fabrication approach produced eutectogels with high conductivity, enhanced mechanical robustness, and excellent biocompatibility. The materials also offered tunable drug release kinetics and high drug loading capacity, presenting clear advantages for controlled transdermal administration. In a subsequent study [100], the same authors developed a dual-layer eutectogel-based MN patch specifically designed for diabetic patients. The MNs were formulated with ChCl, IA, and N-vinyl-2-pyrrolidinone (NVP), and enabled rapid photopolymerization and excellent mechanical strength for effective skin penetration. The microneedle portion contained insulin-loaded nanoparticles for glucose-regulated drug release. The backing layer, composed of a conductive and adhesive eutectogel, containing ChCl, HEMA, glycerol, 3-acrylamidophenylboronic acid (AAPBA), and tannic acid (TA), also demonstrated antibacterial properties, enabling its dual function as a wound dressing and biosensor.

The same research group [101] further advanced this field by developing MNs for wound healing applications inspired by the architecture of crocodile teeth. Incorporating MXene nanosheets within a polymerizable DES matrix (composition details in Table 3) yielded MNs with photothermal responsiveness, antioxidant and anti-inflammatory properties, and sustained drug release capabilities. These MNs promoted wound closure (94.88% closure within 10 days) and inhibited bacterial proliferation (total reduction of bacterial viability by 68%), highlighting their potential for accelerating tissue regeneration. All these examples highlight the versatility of eutectogel-based MNs as well as the advantages of combining the well-known properties of DESs with polymers to obtain improved materials with better rheological properties, bioactivity, and sustained release profiles.

Still in the field of MN production, Ma et al. [96] proposed self-assembled eutectogel-based MNs using an arginine:sorbitol (1:6) NADES without additional polymeric components. The resulting MNs exhibited sharp and uniformly distributed tips, high hydrophilicity, and complete dissolution upon application. They also showed a promising rutin-loading capacity, allowing rutin to permeate mouse skin more than 15-fold higher than that achieved with the rutin solution in vitro.

A different approach using MNs has been reported by Nail et al. [97] where MOF-integrated eutectogel MNs using polymerizable DESs and ZIF-8 were developed to create a UV-curable ink suitable for 3D printing to deliver insulin. The DES formulation contained betaine, methacrylic acid (MAA), and HEMA, in 1:2:2 molar ratio, which was then mixed with polyethylene glycol diacrylate (PEGDA), and the photoinitiator trimethylolpropane triacrylate (T-POL). In vivo studies confirmed the efficient transdermal delivery of insulin, as microneedles applied to diabetic mouse skin created microchannels that gradually closed while the needles dissolved and released the drug. The microneedles progressively disappeared within 3–8 min, demonstrating effective skin penetration, controlled dissolution, and minimal residue. The insulin-loaded MNs achieved sustained, glucose- and pH-responsive release, illustrating the potential of MOF–DES composites in smart transdermal systems.

Finally, in a different study by dos Santos et al. [98], the authors used gelatine methacrylate (GelMA) and PEGDA as the macromolecular matrix, combined with a ChCl:1,2-propanediol (1:2) DES to fabricate mechanically stable MNs capable of effectively penetrating the skin and delivering poorly soluble drugs, such as curcumin, docetaxel, and 5-FU. The results indicated that the used DES effectively improved the solubility of all drugs, with curcumin having the highest solubility, followed by docetaxel, and then 5-FU. The produced MNs showed the expected mechanical properties and were able to penetrate the skin. The release studies also showed a sustained release profile for up to 9 days. These MNs did not exhibit cytotoxic effects towards the fibroblast cell line (NIH/3T3), underscoring their potential for safe and effective transdermal drug delivery.

Overall, there is a growing interest in using eutectogels as platforms for transdermal drug delivery, which offer unique advantages such as enhanced drug solubility, tunable mechanical and release properties, and excellent skin conformability. Their ability to integrate bioactive DES components, form stable interfaces with the skin, and support controlled or stimuli-responsive drug transport positions them as versatile alternatives to conventional hydrogels and ointments. As advances continue in MN design, biocompatible DES selection, and in vivo performance optimization, eutectogels are expected to play an impactful role in the development of next-generation transdermal therapeutic systems.

**Table 3:** Summary table with DES and gelator used, gelation method, and main properties of the eutectogels reported as transdermal delivery systems, including microneedles.

DES	Gelator	Gelation Method	Properties	References
Choline:geranic acid (CAGE, 1:2)	Gelatine	Temperature induced self-assembly combined with dynamical crosslink by tannic acid	Large deformation (400%); Long stress-relaxation times (60% decay after 500 s); Thermo-reversible gel-to-sol phase transitions; Self-adhesion; Stable and robust material	[78]
ChCl:ethylene glycol (1:2)	Calcium ion-coordinated p-hydroxyphenyl methacrylate (HP-Ca^2+^)	Multiple dynamic interactions: double monodentate coordination, strong π–π interaction, double hydrogen bond, and Van der Waals forces	High stretchability (>1600%); Strong tensile strength (>300 kPa); Durable (7 days) and strong skin adhesion (>30 kPa); High temperature tolerance (−25–60 °C)	[92]
Matrine:Lauric acid (3:7)	-	Crystallization in water	Antioxidant; Antibacterial agent; Solvent and stabilizer of curcumin	[93]
ChCl:propylene glycol (1:3)	Tego Carbomer 140 Gelatine	Ionically crosslinked polymer network	Higher drug load and release	[55]
(2-Hydroxypropyl)-β-cyclodextrin:levulinic acid (1:7)	Chitosan	Electrostatic and hydrogen bond crosslinked	In situ film formation; Adhesiveness; Biocompatible; Antioxidant	[94]
Malic acid:ChCl (1:2)	Bassorin fraction (Ba) of Tragacanth gum	Self-assembly	Thermal stability (up to 80 °C); Increase of solubility and permeability of dextromethorphan; Adhesiveness; Resistance to deformation	[95]
ChCl:HEMA:itaconic acid (IA)/glycerol	2-Hydroxyethyl methacrylate (HEMA)	UV-initiated self-crosslinking network	High conductivity; Excellent mechanical strength; Biocompatibility; Tunable drug release kinetics	[99]
**Needle portion (NP):** ChCl, itaconic acid (IA), N-vinyl-2-pyrrolidinone (NVP) (CIN) **Back layer (BL)**: ChCl, HEMA, glycerol, 3-acrylamidophenylboronic acid (AAPBA), and tannic acid (TA) (CHPG)	NP: N-vinyl-2-pyrrolidinone (NVP) BL: HEMA	NP: photopolymerization BL: Hydrogen bonds	Mechanical strength to perforate the skin; Robust; Elongation capacity; Fatigue resistance; Sustained drug release; Adhesiveness	[100]
Vinyl pyrrolidone (VP), itaconic acid (IA), and N-isopropyl acrylamide (NIPAM)	Mxene nanosheets	Photopolymerization-hydrogen bonds	Photothermal response; Temperature-triggered drug release; Antioxidant and anti-inflammatory	[101]
ChCl:1,2-propanediol (1:2)	Gelatine Methacrylate	UV-initiated crosslinking	Improved solubility of APIs; Soft and resistant to deformation; Sustained drug release up to 4 days	[98]
Arginine:sorbitol (1:6)	-	Self-assembly via hydrogen bonding	Excellent mechanical strength; Biocompatibility; High drug loading; Sustained drug release	[96]
Betaine, Methacrylic Acid (MAA), HEMA, Polyethylene Glycol Diacrylate (PEGDA)	ZIF-8	UV-initiated hydrogen bonding	High drug encapsulation and stability Sustained drug release	[97]

### 3.3. Wound Healing and Tissue Engineering

Eutectogels have also demonstrated significant potential in wound healing and tissue engineering applications. Table 4 lists the details of the papers reporting eutectogels developed for these applications. The design of an optimal wound-healing eutectogel must consider multiple factors, namely, providing a sustained antibacterial activity, having enough adhesion to maintain intimate contact without causing trauma upon removal, and providing controlled biodegradability that supports tissue regeneration while avoiding premature degradation [102].

Focusing on wound-healing, Liu et al. [103] developed electroactive poly(deep eutectic solvent)-based eutectogels combined with polysaccharides that allow the production of multifunctional materials with antibacterial and anti-inflammatory properties. The electroactive nature of these gels enables their use in smart wound management systems that integrate treatment with diagnostic functionalities.

In 2025, Wan et al. [104] prepared an eutectogel comprising gelatine and chitosan with the DES betaine:glycerol (1:4). The formulation exhibited suitable rheological properties for wound dressings and demonstrated potent antibacterial activity, enhanced by loading the matrix with chitosan and emodin. In vivo studies revealed accelerated wound healing, reduced bacterial load, and enhanced vascularization and collagen deposition, indicating the potential of eutectogels as multifunctional wound dressings. In a different study, Wang et al. [82] designed a smart cellulose-based eutectogel dressing exhibiting high sensitivity, long-term stability, and excellent antibacterial activity against drug-resistant bacteria, reaching a bacteriostatic rate of 95.7% against *S. aureus* and 82.4% against *E. coli.* Furthermore, the developed formulation was able to promote wound healing rates of 65.6% and 89.1% after 4 and 7 days, respectively, suggesting its suitability for efficient wound healing.

Concerning tissue engineering, Hu et al. [105] reported the synthesis of a highly adhesive eutectogel derived from the DES betaine:citric acid (1:1), combined with *N,N*-dimethylacrylamide (DMAA). The covalent bonding between the polymer matrix and DES components conferred exceptional adhesion, even under elevated temperatures and underwater conditions. The material also exhibited antibacterial activity and excellent biocompatibility towards L929 fibroblasts and red blood cells. Owing to these properties, this eutectogel demonstrated strong potential for applications in tissue adhesion, biosensing, and tissue engineering.

In summary, the application of eutectogels for wound healing and tissue engineering areas keeps growing at a fast pace. The advances in this area are supported by the combination of multiple factors, namely, the biocompatibility, adjustable mechanical properties, and versatility of eutectogels. The possibility to incorporate bioactive DESs, create materials that support cell adhesion and proliferation, modulate inflammatory processes, and even provide the sustained delivery of therapeutic agents makes eutectogels versatile scaffolds for regenerative medicine. Moreover, tuning their antimicrobial, antioxidant, and moisturizing properties enhances their suitability to promote tissue repair and restoration of skin integrity. As research continues to optimize eutectogels’ composition and performance, these materials hold the potential to evolve into clinically relevant platforms that bridge the gap between advanced biomaterials and effective wound management strategies.

## 4. Challenges and Outlook

The field is still in its infancy, with key hurdles including limited structural diversity, solvent loss over time, and trade-offs between conductivity and mechanics. Future work is directed at greener raw materials (e.g., the use of NADESs), scalable processing (3D printing, electrospinning), and multifunctional designs tailored for biomedical and energy applications.

### 4.1. Scalability, Production, and Cost

Although eutectogels can be produced through relatively straightforward mixing and gelation processes, their large-scale production remains a challenge. Maintaining consistent composition, mechanical strength, and solvent retention during upscaling is difficult, especially for formulations sensitive to moisture or temperature. Furthermore, some DES components, particularly functionalized hydrogen-bond donors or acceptors, can be costly or synthetically demanding. Developing standardized, solvent-free, or continuous manufacturing routes could significantly enhance scalability and economic feasibility, paving the way for industrial adoption.

While research on eutectogels’ industrial processing is still scarce, the growing number of patents demonstrates emerging commercial interest. For example, inventors from Shandong Second Medical University [106] describe a photopolymerization-based process for chitosan-modified eutectogels, optimized for adhesion and durability, whereas inventors from Johns Hopkins University [107] protect DES-based gel polymer electrolytes for energy storage devices, both showing that reproducible processing and solvent retention are key for functional applications. In a different patent, Hertel et al. [108] further highlights that choosing inexpensive and abundant DES components (e.g., betaine monohydrate) can improve economic viability. Notably, a recent technology disclosure from McMaster University introduces protein eutectogels designed for extrusion and injection molding, suggesting that thermoplastic eutectogels could be compatible with conventional large-scale manufacturing workflows [109].

Manufacturing processes also face scrutiny since ensuring Good Manufacturing Practice (GMP) compliance for DES-based materials can be challenging due to the lack of standardized production guidelines. DESs are often composed of individual components that are already approved by regulatory agencies such as the FDA or EMA; however, the DES itself, as a newly formed entity, may be classified as a novel excipient and therefore require independent regulatory approval. Furthermore, regulatory pathways for novel hybrid materials such as eutectogels also remain underdeveloped, often requiring a case-by-case assessment which increases time and cost. Collectively, these regulatory hurdles underscore the need for systematic safety studies, standardized characterization methods, and clear regulatory frameworks to enable the broader adoption of eutectogels in biotechnological and clinical settings [110].

### 4.2. Toxicity and Biodegradability

Despite the high potential of eutectogels for biomedical and biotechnological applications, their safety profiles are intricately linked to the specific DES components used and their interactions within the gel matrix.

While individual components such as ChCl, glycerol, and urea are generally considered safe, their eutectic mixtures can exhibit unexpected interactions or altered biological responses. For instance, a study by Macário et al. [75] is a great example of these unexpected results and interactions. The authors tested the cytotoxicity of multiple DESs on human skin cells, and concluded that while some DESs showed low cytotoxicity, others exhibited significant toxicity, underscoring the necessity for comprehensive safety evaluations. Another important aspect is the interactions between DES components and blood components that can influence coagulation, cell lysis, and immune responses, hence hemocompatibility studies are also crucial, especially for applications involving direct contact with blood or implantation. For instance, Mukesh et al. [111] reported that an ion gel, containing an anti-inflammatory drug, formed by self-polymerization of HEMA, was hemocompatible, with the in vitro hemolysis assay showing that there was no dose-dependent hemolysis. More recently, Parsana et al. [83] also reported that the MOF-based eutectogels posed no toxicity towards red blood cells, with hemolysis degrees below the limits determined for toxic response.

### 4.3. Long-Term Stability

The long-term stability of eutectogels remains a major barrier in terms of practical application. These materials can suffer from solvent evaporation, syneresis, or phase separation, especially under unstable environmental conditions. Their application in devices or implants is also limited by mechanical fatigue and loss of ionic conductivity over time. Strategies such as incorporating hydrophobic DESs, introducing covalent or supramolecular crosslinks, or embedding reinforcing nanofillers (e.g., cellulose nanocrystals, silica, or graphene oxide) can be strategical options to overcome these issues. An example of eutectogels designed to have improved stability was described by Lu et al. [54] who demonstrated that eutectogels made from hydrophobic DESs show significantly reduced water uptake (~2.8%) under 90% relative humidity over 30 days. Extending the study up to 1 year allowed the researchers to conclude that, after 365 days, the material retained ~99.3% of its tensile toughness, indicating that with the proper design eutectogels are able to have long-term stability, specifically in adverse conditions of moisture. Using hydrophilic DESs, Meneses et al. [87] (ref) also studied the stability of eutectogels composed of betaine:glycerol and gelatine over 1 year at 37 °C and observed that the gels were stable over the time-period that was studied without significant changes to their rheological properties.

Nonetheless, systematic aging and storage studies are still scarce and urgently needed to guide material optimization.

### 4.4. Opportunities for Hybrid Systems

Hybrid eutectogels [29,86], combining DESs with polymeric, inorganic, or supramolecular components, represent a promising strategy to overcome the inherent limitations of conventional eutectogels [112]. As mentioned in the previous section, eutectogels carry multiple drawbacks, concerning their physical properties, their functional properties, and even the challenges associated with biocompatibility or biodegradability. Consequently, their scalability and processability are also constrained, as it can be difficult to mold, shape, or integrate eutectogels into devices due to low mechanical robustness. Integrating complementary polymers, nanoparticles, or supramolecular networks to obtain hybrid eutectogels can be a promising strategy to improve the materials’ properties. These hybrid eutectogels present enhanced mechanical strength and elasticity, improved solvent retention, and long-term stability. They also introduce new functionalities, tailored biocompatibility and degradation profiles, and enable processing with conventional manufacturing techniques, thereby broadening their applicability in biomedical, environmental, and technological fields [113,114]. Recent studies have demonstrated these advancements; for instance, Jiang et al. [32] developed eutectogels using poly(vinyl) alcohol (PVA), with enhanced mechanical properties through a variable-temperature solvent exchange strategy, while Criado-Gonzalez et al. [115] introduced injectable, conductive, and fluorescent eutectogels with potential for real-time monitoring in biomedical applications.

### 4.5. Do Eutectogels Have a Realistic Future in Real-Life Applications?

**Figure 5:**
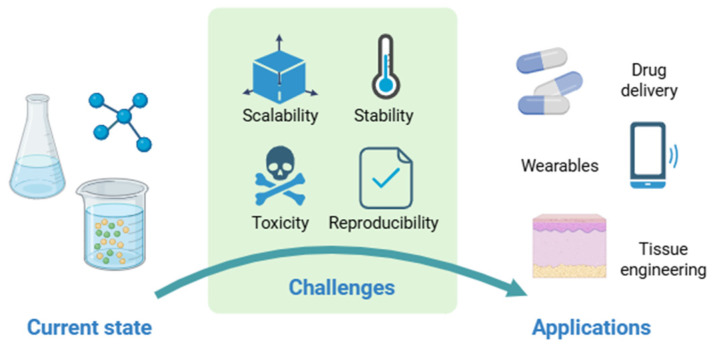
Major challenges of eutectogels in real-life applications (created with BioRender.com).

Standing as a promising intersection between green chemistry and soft materials science, eutectogels have been under the spotlight for their ease of preparation, adjustable physical properties, potential biocompatibility, and versatile applicability. However, achieving their full potential requires addressing current gaps in their development, namely, scalability, reproducibility, stability, and comprehensive toxicity profiling (Figure 5). The progress in this research topic depends on interdisciplinary approaches integrating materials chemistry, bioengineering, and process design. Once these challenges are overcome, eutectogels could evolve from academic curiosities into a new class of sustainable, high-performance soft materials suitable for real-world use.

Confirming the real-life applicability of eutectogels, a wearable monitoring system incorporating eutectogel-based patches for minimally invasive sensing of interstitial fluid in older adults is currently under clinical trial (Tufts University, NCT07217951). While this study does not involve the applications discussed in the previous sections, it illustrates the emerging translational potential of eutectogels in biomedical devices for real-world health monitoring [116].

## Figures and Tables

**Table 4:** Summary table with DES and gelator used, gelation method, and main properties of the eutectogels reported for wound healing and tissue engineering.

DES	Gelator	Gelation Method	Properties	References
Acrylamide (AAm), 2-acrylamido-2 methylpropanesulfonic acid (AMPS), acrylic acid (AAc)	Chitosan	UV-initiated crosslinking	High toughness Fatigue resistance Skin adhesion Good conductivity	[103]
Betaine:glycerol (1:2)	Gelatine Chitosan	Solvent displacement method	Tensile strength (1 MPa) Extensibility (fracture strain > 500%) Antibacterial activity (*S. aureus* and *E. coli*)	[104]
ChCl:glycerol (1:2)	Sodium carboxymethyl cellulose (CMC)	Chemical crosslinking Solvent displacement method	Stretchability (16.83 kPa stress and 190.32% strain) High conductivity (1.14 S/m) Temperature-sensitive Antibacterial activity (*S. aureus*, *E. coli*, MRSA and *P. auruginosa*)	[82]
Betaine:citric acid (1:1)	N-dimethylacetamide (DMAA)	UV-initiated covalent bonds	Good electrical conductivity Mechanical stability High adhesion (under water, extreme temperatures)	[105]

## Data Availability

No new data were created or analyzed in this study. Data sharing is not applicable to this article.

## References

[1] Fiorillo L., Romano G.L. (2020). Gels in Medicine and Surgery: Current Trends and Future Perspectives. Gels.

[2] Rumon M.M.H., Akib A.A., Sarkar S.D., Khan M.A.R., Uddin M.M., Nasrin D., Roy C.K. (2024). Polysaccharide-Based Hydrogels for Advanced Biomedical Engineering Applications. ACS Polym. Au.

[3] Chelu M., Musuc A.M. (2023). Polymer Gels: Classification and Recent Developments in Biomedical Applications. Gels.

[4] Wang H., Du J., Mao Y. (2025). Hydrogel-Based Continuum Soft Robots. Gels.

[5] Meng X., Qi L., Xia C., Jin X., Zhou J., Dong A., Li J., Yang R. (2024). Preparation of Environmentally Friendly, High Strength, Adhesion and Stability Hydrogel Based on Lignocellulose Framework. Int. J. Biol. Macromol..

[6] Khan M.U.A., Aslam M.A., Bin Abdullah M.F., Al-Arjan W.S., Stojanovic G.M., Hasan A. (2024). Hydrogels: Classifications, Fundamental Properties, Applications, and Scopes in Recent Advances in Tissue Engineering and Regenerative Medicine—A Comprehensive Review. Arab. J. Chem..

[7] Wang J., Zhang S., Ma Z., Yan L. (2021). Deep Eutectic Solvents Eutectogels: Progress and Challenges. Green. Chem. Eng..

[8] Mercadal P.A., González A., Beloqui A., Tomé L.C., Mecerreyes D., Calderón M., Picchio M.L. (2024). Eutectogels: The Multifaceted Soft Ionic Materials of Tomorrow. JACS Au.

[9] Joos B., Vranken T., Marchal W., Safari M., Van Bael M.K., Hardy A.T. (2018). Eutectogels: A New Class of Solid Composite Electrolytes for Li/Li-Ion Batteries. Chem. Mater..

[10] Zhang S., Li C. (2025). A Bistate Intelligent Eutectogel Material with Water-Regulated Stiffness for Diverse Designed Functionality. Adv. Funct. Mater..

[11] Shen H., Wang Y., Ma B., Zhou S., Wang J., Yu Z. (2025). An Ultra-Stiff and Tough Glassy Eutectogel. Chem. Commun..

[12] Sun R., Chen D., Li W., Wang B., Hu J. (2025). Highly Sensitive and Environmentally Stable Conductive Eutectogels for Flexible Wearable Sensors Based on Sodium Alginate/Poly(Vinyl Alcohol)/MXene. Int. J. Biol. Macromol..

[13] Nicolau A., Mutch A.L., Thickett S.C. (2024). Applications of Functional Polymeric Eutectogels. Macromol. Rapid Commun..

[14] Datta D., Colaco V., Bandi S.P., Dhas N., Janardhanam L.S.L., Singh S., Vora L.K. (2025). Stimuli-Responsive Self-Healing Ionic Gels: A Promising Approach for Dermal and Tissue Engineering Applications. ACS Biomater. Sci. Eng..

[15] Khalil A.K.A., Teow Y.H., Takriff M.S., Ahmad A.L., Atieh M.A. (2025). Recent Developments in Stimuli-Responsive Polymer for Emerging Applications: A Review. Results Eng..

[16] Abbott A.P., Capper G., Davies D.L., Munro H.L., Rasheed R.K., Tambyrajah V. (2001). Preparation of Novel, Moisture-Stable, Lewis-Acidic Ionic Liquids Containing Quaternary Ammonium Salts with Functional Side Chains. Chem. Commun..

[17] Paiva A., Craveiro R., Aroso I., Martins M., Reis R.L., Duarte A.R.C. (2014). Natural Deep Eutectic Solvents—Solvents for the 21st Century. ACS Sustain. Chem. Eng..

[18] Stephens N.M., Smith E.A. (2022). Structure of Deep Eutectic Solvents (DESs): What We Know, What We Want to Know, and Why We Need to Know It. Langmuir.

[19] Mannu A., Blangetti M., Baldino S., Prandi C. (2021). Promising Technological and Industrial Applications of Deep Eutectic Systems. Materials.

[20] Hansen B.B., Spittle S., Chen B., Poe D., Zhang Y., Klein J.M., Horton A., Adhikari L., Zelovich T., Doherty B.W. (2021). Deep Eutectic Solvents: A Review of Fundamentals and Applications. Chem. Rev..

[21] Joos B., Volders J., Da Cruz R.R., Baeten E., Safari M., Van Bael M.K., Hardy A.T. (2020). Polymeric Backbone Eutectogels as a New Generation of Hybrid Solid-State Electrolytes. Chem. Mater..

[22] Fan K., Wei W., Zhang Z., Liu B., Feng W., Ma Y., Zhang X. (2022). Highly Stretchable, Self-Healing, and Adhesive Polymeric Eutectogel Enabled by Hydrogen-Bond Networks for Wearable Strain Sensor. Chem. Eng. J..

[23] Florindo C., Celia-Silva L.G., Martins L.F.G., Branco L.C., Marrucho I.M. (2018). Supramolecular Hydrogel Based on a Sodium Deep Eutectic Solvent. Chem. Commun..

[24] de Araujo Lima e Souza G., Di Pietro M.E., Mele A. (2023). Eutectic Solvents and Low Molecular Weight Gelators for Next-Generation Supramolecular Eutectogels: A Sustainable Chemistry Perspective. RSC Sustain..

[25] Vanoli V., Pietrowska J., de Araujo Lima e Souza G., Di Pietro M.E., Briatico Vangosa F., Mele A., Castiglione F. (2024). Supramolecular Hydrophobic Eutectogels Based on Menthol-Thymol as Thermo- and PH-Responsive Drug Delivery Systems. ACS Appl. Eng. Mater..

[26] Panzer M.J. (2022). Holding It Together: Noncovalent Cross-Linking Strategies for Ionogels and Eutectogels. Mater. Adv..

[27] Dutta A., Kundu D., Naik P.K., Silvester D.S., Banerjee T. (2024). Synthesis and Properties of Physically Cross-Linked Silica-Mediated Novel Eutectogels Developed from Carboxylic Acid-Based Natural Deep Eutectic Solvents. Ind. Eng. Chem. Res..

[28] Zhang X., Li X., Yang J., Liu Y. (2025). Stretchable, Fatigue-Resistant, and Temperature-Tolerant Multifunctional Dual-Network Eutectogel Based on Metal Salt-Based Deep Eutectic Solvent for Strain Sensors and Triboelectric Nanogenerators. Chem. Eng. J..

[29] Gu B., Qin W., Li G., Ji H., Huang B., Lin B., Xu C., Wei Y., Fu L. (2025). Tough, Strongly Adhesive, and Highly Conductive Eutectogels Enabled by Homogeneous and Stable Organic–Inorganic Hybrid Networks. Chem. Eng. J..

[30] Ding K., Ning H., Liu H., Zhang X., Zhao Y., Zhang S. (2025). Preparation, Properties, and Applications of Eutectogels. J. Inorg. General. Chem..

[31] Mota-Morales J.D., Sánchez-Leija R.J., Carranza A., Pojman J.A., del Monte F., Luna-Bárcenas G. (2018). Free-Radical Polymerizations of and in Deep Eutectic Solvents: Green Synthesis of Functional Materials. Prog. Polym. Sci..

[32] Jiang Y., Tang N., Wang X., Pei D., Zhang H., Li M.H., Hu J. (2025). Mechanically Robust Eutectogels Enabled by Precisely Engineered Crystalline Domains. Nat. Commun..

[33] Lu Q., Li H., Tan Z. (2024). Physically Entangled Multifunctional Eutectogels for Flexible Sensors with Mechanically Robust. J. Mater. Chem. A Mater..

[34] Rajawasam C.W.H., Dodo O.J., Weerasinghe M.A.S.N., Raji I.O., Wanasinghe S.V., Konkolewicz D., De Alwis Watuthanthrige N. (2023). Educational Series: Characterizing Crosslinked Polymer Networks. Polym. Chem..

[35] Weerasinghe U.A., Wu T., Chee P.L., Yew P.Y.M., Lee H.K., Loh X.J., Dan K. (2024). Deep Eutectic Solvents towards Green Polymeric Materials. Green Chem..

[36] Vo T.H., Lam P.K., Sheng Y.J., Tsao H.K. (2025). A Functional Eutectogel Based on Ultrahigh–Molecular Weight Polymers: Physical Entanglements in Deep Eutectic Solvent. J. Colloid Interface Sci..

[37] Liao S., Qu J. (2025). Self-Adhesive Wearable Strain Sensors Based on Transparent, Tough, Antibacterial, Self-Healing Polymeric Eutectogels. ACS Appl. Polym. Mater..

[38] Liang Y., Zou D., Zhang Y., Zhong Z. (2023). Indirect Method for Preparing Dual Crosslinked Eutectogels with High Strength, Stretchability, Conductivity and Rapid Self-Recovery Capability as Flexible and Freeze-Resistant Strain Sensors. Chem. Eng. J..

[39] Yu K., Gao Y., Wang R., Wu L., Ma X., Fang Y., Fang X., Dou Q. (2024). Ultra-Tough and Highly Stretchable Dual-Crosslinked Eutectogel Based on Coordinated and Non-Coordinated Two Types Deep Eutectic Solvent Mixture. Macromol. Rapid Commun..

[40] Qin H., Panzer M.J. (2017). Chemically Cross-Linked Poly(2-Hydroxyethyl Methacrylate)-Supported Deep Eutectic Solvent Gel Electrolytes for Eco-Friendly Supercapacitors. ChemElectroChem.

[41] Hong S., Yuan Y., Liu C., Chen W., Chen L., Lian H., Liimatainen H. (2020). A Stretchable and Compressible Ion Gel Based on a Deep Eutectic Solvent Applied as a Strain Sensor and Electrolyte for Supercapacitors. J. Mater. Chem. C Mater..

[42] Mota-Morales J.D., Gutiérrez M.C., Ferrer M.L., Jiménez R., Santiago P., Sanchez I.C., Terrones M., Del Monte F., Luna-Bárcenas G. (2013). Synthesis of Macroporous Poly(Acrylic Acid)-Carbon Nanotube Composites by Frontal Polymerization in Deep-Eutectic Solvents. J. Mater. Chem. A Mater..

[43] Li R., Chen G., Fan T., Zhang K., He M. (2020). Transparent Conductive Elastomers with Excellent Autonomous Self-Healing Capability in Harsh Organic Solvent Environments. J. Mater. Chem. A Mater..

[44] Isik M., Ruiperez F., Sardon H., Gonzalez A., Zulfiqar S., Mecerreyes D. (2016). Innovative Poly(Ionic Liquid)s by the Polymerization of Deep Eutectic Monomers. Macromol. Rapid Commun..

[45] Jesus A.R., Meneses L., Duarte A.R.C., Paiva A. (2021). Natural Deep Eutectic Systems, an Emerging Class of Cryoprotectant Agents. Cryobiology.

[46] Tian Y., Sun D.W., Zhu Z. (2022). Development of Natural Deep Eutectic Solvents (NADESs) as Anti-Freezing Agents for the Frozen Food Industry: Water-Tailoring Effects, Anti-Freezing Mechanisms and Applications. Food Chem..

[47] Heydari F., Mohamadnia Z. (2025). Self-Healing, Ultra Stretchable, Antifreezing, and Antidrying Gelatin-Based Eutectogels Containing Polymerizable Deep Eutectic Solvent for Advanced Motion Sensing. ACS Appl. Polym. Mater..

[48] Hu X., Zhao Y., Pu L., Chu X., Sun C., Liu H. (2024). Stretchable Anti-Freeze Deep Eutectic Solvent (DES) Gels for Low-Temperature Wearable Soft Sensors. New J. Chem..

[49] Reuter D., Binder C., Lunkenheimer P., Loidl A. (2019). Ionic Conductivity of Deep Eutectic Solvents: The Role of Orientational Dynamics and Glassy Freezing. Phys. Chem. Chem. Phys..

[50] Lyu G., Korte C., Luo J. (2025). Deep Eutectic Solvents Based on N-Methyltrifluoroacetamide and Lithium Bis(Trifluoromethanesulfonyl)Imide as New Electrolytes with Low Viscosity and High Ionic Conductivity. Materials.

[51] Yang J., Chang L., Deng H., Cao Z. (2024). Zwitterionic Eutectogels with High Ionic Conductivity for Environmentally Tolerant and Self-Healing Triboelectric Nanogenerators. ACS Nano.

[52] Huang Z., Xie J., Li T., Xu L., Liu P., Peng J. (2024). Highly Transparent, Mechanically Robust, and Conductive Eutectogel Based on Oligoethylene Glycol and Deep Eutectic Solvent for Reliable Human Motions Sensing. Polymers.

[53] Nechausov S., Jiang Y., Miriyev A. (2025). I-Wi: Multifunctional 3D-Printable Stretchable Ionogel and Ionic Eutectogel Wires with AC and DC Signal Transmission. Addit. Manuf..

[54] Lu Q., Li H., Cai C., Tan Z. (2025). Hydrophobic Deep Eutectic Solvent-Based Eutectogels for Long-Term Humidity Resistance and Multifunctional Sensing. Mater. Horiz..

[55] Cassano R., Sole R., Siciliano C., Baldino N., Mileti O., Procopio D., Curcio F., Calviello G., Serini S., Trombino S. (2024). Eutectogel-Based Drug Delivery: An Innovative Approach for Atenolol Administration. Pharmaceutics.

[56] Kim M.J., Cho S.H., Oh S.J., Kim S.W. (2025). Ultrastretchable, Fatigue-Resistant Eutectogel with Hierarchical Bonding for Advanced Wearable Monitoring. Adv. Compos. Hybrid. Mater..

[57] Wang J., Zhen Y., Hao J., Chai C. (2025). Recent Advances in Eutectogels: Preparation, Properties and Applications. Adv. Colloid Interface Sci..

[58] Ejeromedoghene O., Orege J.I., Oderinde O., Okoye C.O., Alowakennu M., Nnyia M.O., Fu G. (2022). Deep Eutectic Solvent-Assisted Stimuli-Responsive Smart Hydrogels—A Review. Eur. Polym. J..

[59] Vicente F.A., Tkalec N., Likozar B. (2024). Responsive Deep Eutectic Solvents: Mechanisms, Applications and Their Role in Sustainable Chemistry. Chem. Commun..

[60] Huo X., Wang J., Cong Z., Liu C., Cai C., Wang Y., Zhang X., Li C., Lan S., Niu J. (2025). Strong and Tough Eutectogels with Broad-Range Tunable Mechanical Properties via the Hydrogen Bond Network-Specific Effect. Adv. Funct. Mater..

[61] Chen S., Feng J. (2023). Facile Solvent Regulation for Highly Strong and Tough Physical Eutectogels with Remarkable Strain Sensitivity. ACS Appl. Mater. Interfaces.

[62] Arjunan K.K., Weng C.Y., Sheng Y.J., Tsao H.K. (2024). Formation of Self-Healing Granular Eutectogels through Jammed Carbopol Microgels in Supercooled Deep Eutectic Solvent. Langmuir.

[63] Li M., Liu Z., Hu Y., Li R., Cao Y. (2023). A Hydrophobic Eutectogel with Excellent Underwater Self-Adhesion, Self-Healing, Transparency, Stretchability, Ionic Conductivity, and Fully Recyclability. Chem. Eng. J..

[64] Yang M., Cao Z., Qiu X., Zhou Y., Liu L., Yang W., Yao J. (2025). Mechanically Robust, Temperature Tolerant, Adhesive, Self-Healing, and Transparent Supramolecular Eutectogels as Flexible Sensors. ChemistrySelect.

[65] Cao Q., Yuan W. (2025). Photochromic Hydrophobic Eutectogel with High Adhesion, Stability, and Self-Healing for Information Encryption, Underwater Wireless Communications, and Motion Monitoring. Nano Micro Small.

[66] Zhang Z., Yao A., Raffa P. (2025). Physically Crosslinked Amphiphilic Eutectogels with Underwater Self-Healing, Strong Adhesion, Environmental Stability and Closed-Loop Recyclability for Underwater Sensing and Information Transmission. Chem. Eng. J..

[67] Padhan A., Singh V. (2025). Nature Mimicking Temperature Induced Self-Healing Soft Subaquatic Superoleophobic Polymeric Materials for Oil-Water Separation. J. Mol. Liq..

[68] Kalidindi S., Zackin A.K., Alsaedi M.K., Sharma A., Zeng W., Owyeung R.E., Kang H., Sonkusale S., Panzer M.J., Yi H. (2025). Facile Evaporation-Stamping Technique for Stimuli-Responsive Biopolymer-Supported Eutectogels Containing Opal Micropatterns. ACS Appl. Mater. Interfaces.

[69] Prasad K., Mondal D., Sharma M., Freire M.G., Mukesh C., Bhatt J. (2018). Stimuli Responsive Ion Gels Based on Polysaccharides and Other Polymers Prepared Using Ionic Liquids and Deep Eutectic Solvents. Carbohydr. Polym..

[70] Parsana N., Ukani H., El Seoud O.A., Al-Ghamdi A., Malek N. (2024). Deep Eutectic Solvent Based Self-Healable, Stretchable and Injectable Eutectogels: A Versatile Platform for Breast Cancer Treatment. Chem. Eng. J..

[71] Nining N., Wardhana Y.W., Rusdiana T., Hariyanti H. (2025). Deep Eutectic Solvents in Polymeric Drug Carriers: Insights into Release Behavior and Functional Integration. ChemistryOpen.

[72] Jesus A.R., Paiva A., Duarte A.R.C. (2023). Current Developments and Future Perspectives on Biotechnology Applications of Natural Deep Eutectic Systems. Curr. Opin. Green Sustain. Chem..

[73] Joarder S., Bansal D., Meena H., Kaushik N., Tomar J., Kumari K., Bahadur I., Ha Choi E., Kaushik N.K., Singh P. (2023). Bioinspired Green Deep Eutectic Solvents: Preparation, Catalytic Activity, and Biocompatibility. J. Mol. Liq..

[74] Ferreira I.J., Paiva A., Diniz M., Duarte A.R. (2023). Uncovering Biodegradability and Biocompatibility of Betaine-Based Deep Eutectic Systems. Environ. Sci. Pollut. Res..

[75] Macário I.P.E., Oliveira H., Menezes A.C., Ventura S.P.M., Pereira J.L., Gonçalves A.M.M., Coutinho J.A.P., Gonçalves F.J.M. (2019). Cytotoxicity Profiling of Deep Eutectic Solvents to Human Skin Cells. Sci. Rep..

[76] Güvenalp N., Güvenç D. (2020). An Evaluation of the Effects of Medium PH on the Viability of the HepG2 Cell Line. Etlik Vet. Mikrobiyoloji Derg..

[77] Xia H., Ren M., Zou Y., Qin S., Zeng C. (2020). Novel Biocompatible Polysaccharide-Based Eutectogels with Tunable Rheological, Thermal, and Mechanical Properties: The Role of Water. Molecules.

[78] Picchio M.L., Orellano M.S., Motta M.A., Huck-Iriart C., Sánchez-deAlcázar D., López-Domene R., Martín-García B., Larrañaga A., Beloqui A., Mecerreyes D. (2024). Elastomeric Protein Bioactive Eutectogels for Topical Drug Delivery. Adv. Funct. Mater..

[79] Filip D., Macocinschi D., Balan-Porcarasu M., Varganici C.D., Dumitriu R.P., Peptanariu D., Tuchilus C.G., Zaltariov M.F. (2022). Biocompatible Self-Assembled Hydrogen-Bonded Gels Based on Natural Deep Eutectic Solvents and Hydroxypropyl Cellulose with Strong Antimicrobial Activity. Gels.

[80] Bianchi M.B., Zhang C., Catlin E., Sandri G., Calderón M., Larrañeta E., Donnelly R.F., Picchio M.L., Paredes A.J. (2022). Bioadhesive Eutectogels Supporting Drug Nanocrystals for Long-Acting Delivery to Mucosal Tissues. Mater. Today Bio.

[81] Mercadal P.A., Romero M.R., del Montesinos M.M., Real J.P., Picchio M.L., González A. (2023). Natural, Biocompatible, and 3D-Printable Gelatin Eutectogels Reinforced with Tannic Acid-Coated Cellulose Nanocrystals for Sensitive Strain Sensors. ACS Appl. Electron. Mater..

[82] Wang Z., Qi B., Wang R., Chen Z., Zhong J., Wang Z., Sun Q., Yu A., Shen X., Xie H. (2025). Smart Eutectogel with Antibacterial Activity for Efficiently Treating Multidrug Resistant Infection, Real-Time Monitoring and Diabetic Wound Repair. Chem. Eng. J..

[83] Parsana N., Ukani H., Chauhan D.S., El Seoud O., Mehra S., Kumar A., Raje N., Malek N. (2024). Biocompatible, Injectable and Self-Healable MOF-Based Anti-Freezing Eutectogels for Higher Encapsulation and Sustained Release of the Anticancer Drug Curcumin. RSC Pharm..

[84] Shaw Z.L., Awad M.N., Gharehgozlo S., Greaves T.L., Haidari H., Kopecki Z., Bryant G., Spicer P.T., Walia S., Elbourne A. (2024). Deep Eutectic Solvent Eutectogels for Delivery of Broad-Spectrum Antimicrobials. ACS Appl. Bio Mater..

[85] Albertini B., Bertoni S., Nucci G., Botti G., Abrami M., Sangiorgi S., Beggiato S., Prata C., Ferraro L., Grassi M. (2024). Supramolecular Eutectogel as New Oral Paediatric Delivery System to Enhance Benznidazole Bioavailability. Int. J. Pharm..

[86] Anuța V., Nica M.A., Prisada R.M., Popa L., Velescu B.Ș., Marinas I.C., Gaboreanu D.M., Ghica M.V., Cocoș F.I., Nicolae C.A. (2025). Novel Buccal Xanthan Gum–Hyaluronic Acid Eutectogels with Dual Anti-Inflammatory and Antimicrobial Properties. Gels.

[87] Meneses L., Bagaki D.A., Roda A., Paiva A., Duarte A.R.C. (2024). Development of Enzymatically Crosslinked Natural Deep Eutectogels: Versatile Gels for Enhanced Drug Delivery. J. Mater. Chem. B.

[88] Cheng Z., Kang M., Peng X., Ren L., Xie J., Yuan Q., Xu X., Li J. (2025). Self-Assembled Eutectogel with Cell Permeation and Multiple Anti-Inflammatory Abilities for Treating Chronic Periodontitis. Adv. Mater..

[89] Chachad J., Dey S., Sherje A., Dangre P., Borkar M.R. (2025). Fabrication of Sodium Alginate-Hydroxypropyl Methylcellulose Based Bioadhesive Eutectogel of Eberconazole for Improved Anti-Fungal Activity. Carbohydr. Polym..

[90] Yuan J., Liu Z., Yin T., Meng S. (2025). A Carrier-Free Therapeutic Eutectogel: Construction, Physical Properties, and Anti-Bacterial Ability. RSC Adv..

[91] Bryant S., Elbourne A., Walia S., Awad M., Bryant G., Greaves T. (2025). Antimicrobial Gels.

[92] Zhang Y., Liu C., Zhang S., Li J., Quan P., Song Y., Liu J., Fang L. (2023). Multiple Dynamic Interaction-Enabled Eutectogel with Strong Tissue Adhesion, Mechanical Strength and Temperature Tolerance for Transdermal Drug Delivery: Double Monodentate Coordination and π-π Interaction. Chem. Eng. J..

[93] Yang Q., Liu Z., Yin T., Wang X., Yuan J. (2024). Eutectogel Based on Multi-Functional Deep Eutectic Solvent for Acne Infection Treatment. J. Drug Deliv. Sci. Technol..

[94] Li B., Li X., Li S., Chen C., Xiao T., Xu Y. (2025). Supramolecular Cyclodextrin Deep Eutectic Solvent-Strengthened Chitosan Eutectogel as a Novel Percutaneous Delivery System of Resveratrol for Anti-Psoriasis. Int. J. Biol. Macromol..

[95] Fatahi A., Varshosaz J., Hajhashemi V. (2025). Tragacanth Gum-Deep Eutectic Solvent-Based Eutectogels for Dextromethorphan Transdermal Delivery and Induced Rheumatoid Arthritis Treatment in Rat. Int. J. Biol. Macromol..

[96] Ma H., Li Y., Yao Q., Qin R., Wang Y., Wu W., Luo H., Zhao Q., Ye H., Wu K. (2025). Self-Assembled Arginine-Based Eutectogel Microneedles as Novel Transdermal Delivery System for Pigmentation Treatment. Giant.

[97] Nail A., Liu H., Meng D., Zhu L., Guo X., Li C., Yu H., Yu Z., Li H. (2025). 3D-Printed Insulin/GOx-Loaded ZIF-8 Microneedles via Hydrogen-Bond-Enhanced Photopolymerization for Transdermal Drug Delivery. Surf. Interfaces.

[98] dos Santos D.M., Suresh H., Kruzshak S.J., Kim J., Cebe P., Baleja J.D., Tzanakakis E.S., Sonkusale S. (2025). Engineering Eutectogel Microneedle Patch as Effective Transdermal Delivery System of Hydrophobic Drugs. Adv. Ther..

[99] Liu H., Zhou X., Nail A., Yu H., Yu Z., Sun Y., Wang K., Bao N., Meng D., Zhu L. (2024). Multi-Material 3D Printed Eutectogel Microneedle Patches Integrated with Fast Customization and Tunable Drug Delivery. J. Control. Release.

[100] Liu H., Nail A., Meng D., Zhu L., Guo X., Li C., Li H. (2025). 3D Printed Eutectogel Dissolving Microneedles Patch Loaded with Chitosan-Based Nanoparticles for Diabetic Wound Management. Int. J. Biol. Macromol..

[101] Liu H., Nail A., Meng D., Zhu L., Guo X., Li C., Ye X., Li H. (2025). Bioinspired 3D-Printed NIR-Responsive MXene-Based Multifunctional Eutectogel Microneedles for Personalized Infected Wound Healing. Adv. Healthc. Mater..

[102] Baniasadi H. (2025). State-of-the-Art in Natural Hydrogel-Based Wound Dressings: Design, Functionalization, and Fabrication Approaches. Adv. Colloid Interface Sci..

[103] Liu S., Zhan J., Liu Z., Tan X., Huang J., Pu C., Lin R., Chen Y., Luo Q., Qiu X. (2025). Versatile Poly (Deep Eutectic Solvents) Electroactive Chitosan Eutectogel for Infected Wound Healing and Monitoring Administration. Carbohydr. Polym..

[104] Wan J., Yin M., Wang F., Huang G., Pra I.D., Liang Y., Wu J. (2025). Natural Gelatin / Chitosan Oligosaccharides / Emodin Eutectogels with Multiple Antibacterial Effects for Accelerated Infected Wound Healing. Int. J. Biol. Macromol..

[105] Hu Y., Zhan J., Pu C., Yang W., Zhong L., Xu Z., Pan Y., Hou H. (2025). Multi-Functional Covalent Polymer Eutectogels Based on Deep Eutectic Solvents Potential for Sealable Adhesive. Chem. Eng. J..

[106] Tang S. (2024). Preparation Method of Deep Eutectic Liquid Gel and Its Products and Applications.

[107] Gerasopoulos K., Hoffman C.M., Freeman A.W., Logan M.W., Langevin S.A. (2019). Deep Eutectic Solvent-Based Gel Polymer Electrolytes.

[108] Hertel R.M., Bommarius A.S., Realff M.J., Kang Y. (2012). Deep Eutectic Solvent Systems and Methods.

[109] Shanbhag S., Selvagnapathy P.R. Protein Eutectogel as a Platform for Advanced Materials and Manufacturing. https://research.mcmaster.ca/industry-investors/tech/23-085/.

[110] Kozarewicz P., Loftsson T. (2018). Novel Excipients—Regulatory Challenges and Perspectives—The EU Insight. Int. J. Pharm..

[111] Mukesh C., Upadhyay K.K., Devkar R.V., Chudasama N.A., Raol G.G., Prasad K. (2016). Preparation of a Noncytotoxic Hemocompatible Ion Gel by Self-Polymerization of HEMA in a Green Deep Eutectic Solvent. Macromol. Chem. Phys..

[112] Kumar K., Calderón M., Beloqui A., Picchio M.L. (2024). Eutectogels as Promising Materials in Biocatalysis. ChemCatChem.

[113] Yang J., Feng Y., Wang B., Miao J., Wei S., Li H., Mo L., Qin Z. (2023). Tough, Multifunctional, and Green Double-Network Binary Solvent Eutectogel with in-Situ Generation of Lignin Nanoparticles Based on One-Step Dual Phase Separations for Wearable Flexible Strain Sensors. Chem. Eng. J..

[114] Zhang B., Sun H., Huang Y., Zhang B., Wang F., Song J. (2021). Multifunctional Supramolecular Eutectogels for Self-Healable Conductive Materials and Interface Lubrication. Chem. Eng. J..

[115] Criado-Gonzalez M., Alegret N., Fracaroli A.M., Mantione D., Guzmán-González G., Del Olmo R., Tashiro K., Tomé L.C., Picchio M.L., Mecerreyes D. (2023). Mixed Conductive, Injectable, and Fluorescent Supramolecular Eutectogel Composites. Angew. Chem. Int. Ed..

[116] Tufts University Assessing the Feasibility of Multi-Modal Biosensing for Monitoring Mobility and Cognition in Older Adults. https://clinicaltrials.gov/study/NCT07217951?term=eutectogel&rank=1&tab=history.

